# Inflammatory cutaneous lesions and pulmonary manifestations in a new patient with autosomal recessive ISG15 deficiency case report

**DOI:** 10.1186/s13223-020-00473-7

**Published:** 2020-09-03

**Authors:** Guadalupe Buda, Rita María Valdez, German Biagioli, Federico A. Olivieri, Nicolás Affranchino, Carolina Bouso, Vanesa Lotersztein, Dusan Bogunovic, Jacinta Bustamante, Marcelo A. Martí

**Affiliations:** 1 Departamento de Química Biológica, Facultad de Ciencias Exactas y Naturales, Universidad de Buenos Aires (FCEyN-UBA) e Instituto de Química Biológica de la Facultad de Ciencias Exactas y Naturales (IQUIBICEN) CONICET, Pabellón 2 de Ciudad Universitaria, Buenos Aires, Argentina; 2 Bitgenia, Buenos Aires, Argentina; 3 Hospital Militar Central, Servicio de Genética, Buenos Aires, Argentina; 4 Hospital Juan P. Garrahan, Servicio de Pediatría, Buenos Aires, Argentina; 5 Hospital Juan P. Garrahan, Servicio de Inmunología y Reumatología, Buenos Aires, Argentina; 6 Department of Microbiology, Icahn School of Medicine at Mount Sinai, New York, USA; 7 Department of Pediatrics, Icahn School of Medicine at Mount Sinai, New York, USA; 8 The Mindich Child Health and Development Institute, Icahn School of Medicine at Mount Sinai, New York, USA; 9 Laboratory of Human Genetics of Infectious Diseases, Necker Branch, INSERM, Paris, France; 10 Laboratory of Human Genetics of Infectious Diseases, Necker Branch, INSERM, University of Paris, Imagine Institute, Paris, EU France; 11 St. Giles Laboratory of Human Genetics of Infectious Diseases, Rockefeller Branch, The Rockefeller University, New York, USA; 12 Study Center of Immunodeficiencies, Necker Hospital for Sick Children, Paris, EU France

**Keywords:** Whole-exome sequencing, *ISG15* gene, Case report, Ulcerative skin lesions, Lung disease

## Abstract

Interferon-stimulated gene 15 (*ISG15)* was the first ubiquitin-like modifier protein identified that acts by protein conjugation (ISGylation) and is thought to modulate IFN-induced inflammation. Here, we report a new patient from a non-consanguineous Argentinian family, who was followed for recurrent ulcerative skin lesions, cerebral calcifications and lung disease. Whole Exome Sequencing (WES) revealed two novel compound heterozygous variants (c.285del and c.299_312del, NM_005101.4 GRCh37(hg19), both classified as pathogenic according to ACMG criteria) in the *ISG15* gene, resulting in a complete deficiency due to disruption of the second ubiquitin domain of the corresponding protein. The clinical phenotype of this patient is unique given the presence of recurrent pulmonary manifestations and the absence of mycobacterial infections, thus resulting in a phenotype distinct from that previously described in patients with biallelic loss-of-function (LOF) *ISG15* variants. This case highlights the role of *ISG15* as an immunomodulating factor whose LOF variants result in heterogeneous clinical presentations.

## To the Editor

Interferon-stimulated gene 15 *(ISG15*) was the first ubiquitin-like modifier protein identified that acts by protein conjugation (ISGylation) [1]. It is one of the most upregulated genes upon Type I interferon treatment or pathogen infections, and its secreted form stimulates the production of cytokines and proliferation of NK cells in humans [2]. ISG15 is present in the gelatinase and secretory granules (but not in the azurophilic or specific granules) of steady-state neutrophils, which release it upon bacterial challenge [3]. ISG15 is also secreted by many other cell types, including myeloid cells, acting as a very potent interferon gamma (IFN-γ) inducing cytokine in lymphocytes (particular NK cells), and in synergy with IL-12. In humans, biallelic mutations in *ISG15* were reported to result in an impaired IFN-γ mediated immunity, leading to an increase predisposition to mycobacterial infections in most cases, as also observed in patients with Mendelian Susceptibility to Mycobacterial Diseases (MSMD) [4]; [5]; [6] (see Additional file 1: Table S2). Interestingly, ISG15-deficient patients usually present features of type 1 interferonopathy with cerebral calcifications; attributed to the role of ISG15 in controlling IFN-alpha/beta responses [8].

**Fig. 1:**
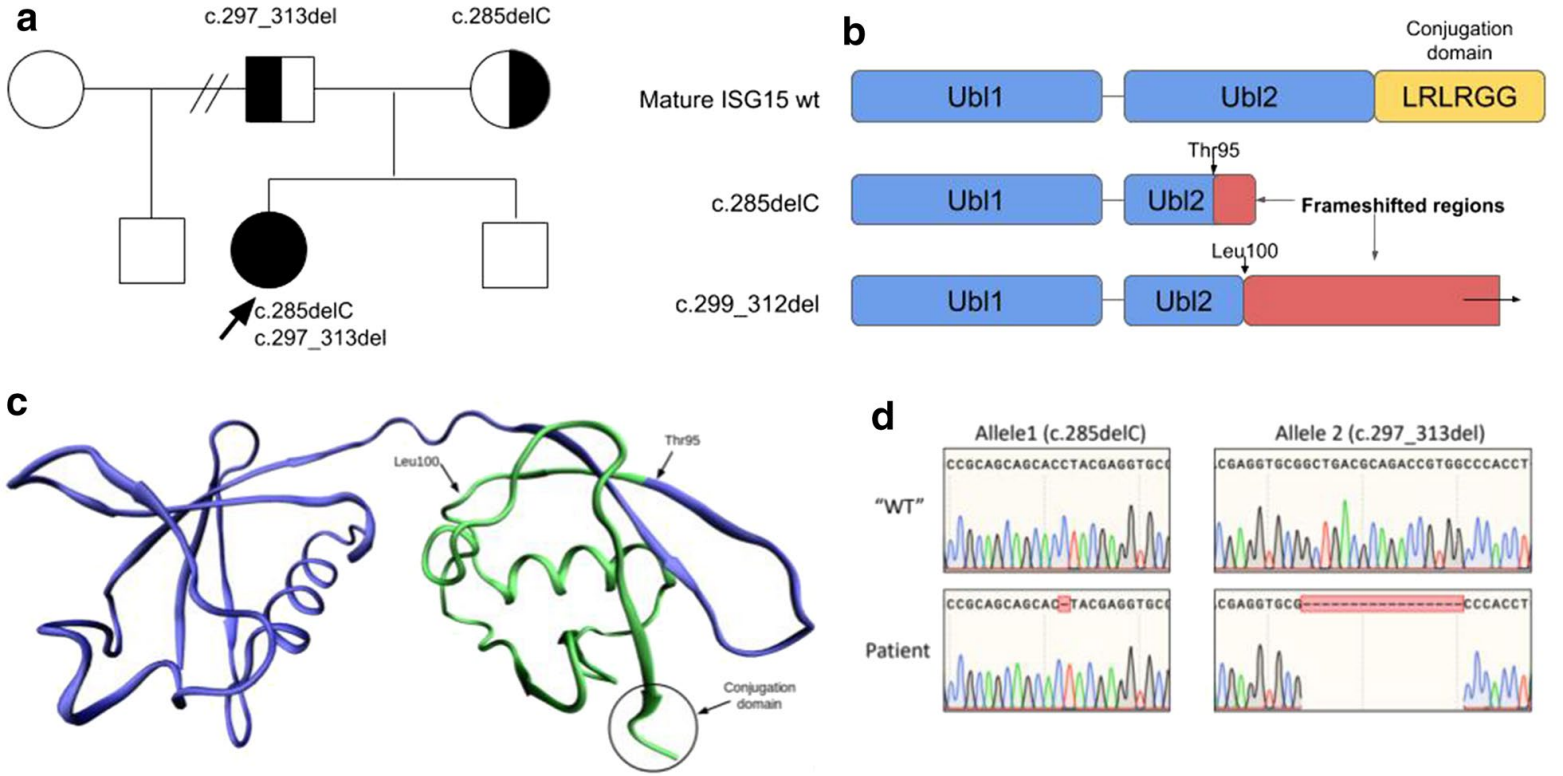
**a** *Family pedigree.* The proband is indicated by an arrow (P). The segregation was validated by Sanger Sequencing in the main family. **b** *ISG15 consists of two ubl domains (blue) that are linked by a proline residue.* ISG15 is synthesized as a 17-kDa precursor that is proteolytically processed into a mature form of 15 kDa. This processing exposes a carboxy-terminal LRLRGG motif, required for ISGylation (yellow). The c.285delC variant produces a frameshift after Threonine 95 which results in a stop codon downstream. The c.299_312del produces a frameshift after Leucine 100 that has no stop codon within the sequence of the original gene. **c** *Crystallographic structure of human ISG15.* The area disabled by the mutations is marked in green. Thr95 marks the beginning of the 285_del frameshift. Leu100 marks the beginning of the 299_312del frameshift. The C-terminus of the structure corresponds to the first 3 residues of the conjugation domain. **d** *Partial sequence chromatograms for ISG15 in the patient.* DNA sequences of the WT (wild-type) and mutant *ISG15* alleles are shown. Red rectangle denotes the sites of heterozygous sequence deletions

In the present work, we report a new patient with autosomal recessive (AR) complete *ISG15* deficiency, followed for ulcerative skin lesions and lung disease. The patient was born in 2013 from a non-consanguineous Argentinean family. The brotherhood is completed with a healthy younger brother, and a healthy paternal stepbrother (Fig. 1a). She received BCG (**B**acillus **C**almette–**G**uérin) vaccination at birth. At 6 months of age, she presented with neck and axillary papulo-ulcerated lesions. At that time she was admitted at the hospital and received antibiotic treatment without clinical response, followed by spontaneous resolution 2 months later. Between 6 and 15 months of age, and due to recurrent skin ulcers, she was initially suspected of presenting BCG-itis. At 1 year old she showed novel similar lesions in the inguinal region and external genitalia, with bilateral and asymmetrical location and without response to either local or systemic treatments. The lesions showed slow healing (3 months) and the biopsy reported ulceration of dermis and epidermis, with necrosis of underlying hypodermis and without inflammatory reaction. Periodic acid–Schiff, acid-fast bacilli and Ziehl–Neelsen reactions were negative. Cultures for pyogenic bacteria, mycobacteria and fungi were also negative. Immunofluorescence of skin samples were negative for immunoglobulins (Ig) IgA, IgG, IgM and C3.

**Fig. 2:**
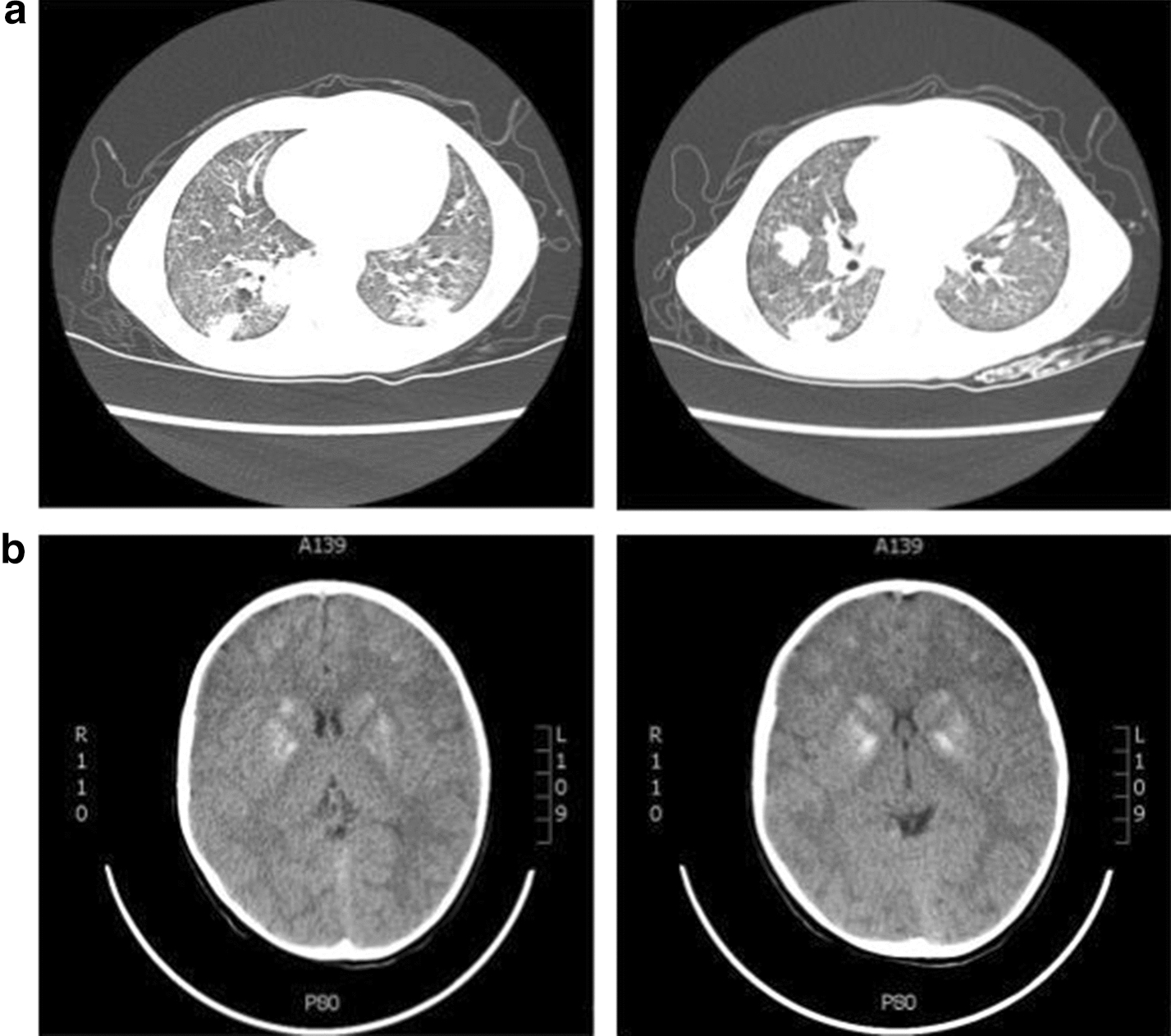
**a** Chest CT scan. Bilateral focal areas of parenchymal consolidations, some of them with air bronchograms and with nodular appearance, distributed in the peripheral portions of the lower zones. Bilateral widespread septal thickening and peripheral tree in-bud changes in both lungs is observed too. Lobar air-trapping (ILL) on expiration was present. **b** Brain CT scan. Calcifications at the level of the caudate nuclei, lenticular nuclei and subcortical region of both frontal lobes are shown in the images

Starting at 2 years and 8 months of age, the patient began having respiratory manifestations including recurrent wheeze and lobar pneumonias (seven isolated and unrelated episodes over the span of 4 years). Three of them required hospitalization, antibiotics and oxygen therapy (for a period of 5 days on average); while one presented with images resembling necrotizing pneumonia with negative microbiological tests. She was kept asymptomatic between episodes. Lung and chest computed tomography (CT) scanning (Fig. 2a) showed some non specific and residual lesions, while brain CT evidenced basal ganglia calcifications (Fig. 2b). Due to the pulmonary manifestations, mycobacterial infection was suspected again. However, clinical inspection by the attending physician discarded BCG-itis, since: (i) no mycobacteria species (or other microorganisms) were isolated from either skin biopsies, or from multiple bronchoalveolar lavages (BAL); (ii) the skin lesions healed without the use of any antimycobacterial treatment, and (iii) the pulmonary manifestations, although seemed progressive, were presented in multiple episodes affecting different sections of the lung parenchyma.

Laboratory tests showed elevated acute-phase reactants, mainly the erythrocyte sedimentation rate. Number and percentages of T, B and NK cells showed to be in adequate range for the age, as well as Igs, C3 and C4. In addition, inadequate antibody response to some protein antigens and polysaccharides was found (Additional file 3: Table S3). The dihydrorhodamine (DHR) assay on neutrophils was normal. Autoimmune workup revealed transient positive lupus anticoagulant and non specific ASMA auto-Ab. Due to the patient multisystemic picture affecting predominantly the skin (chronic and recurrent ulcers) and the lower respiratory tract (lung disease associated with hypoxia and persistent lesions despite adequate treatment), the patient was initially suspected to carry an autoinflammatory disease. Currently, the patient is 7 years old and presents height and weight appropriate for her age. Healing lesions are still present in retroauricular regions, cervical, bilateral axillary, bilateral inguinal and in labia majora, with associated lipoatrophy. She shows delayed and peculiar dentition with the absence of canines and no other abnormalities. Additionally, she displays chronic lung disease with hypoxemic chronic respiratory failure and is being treated with oxygen therapy at home (0.5 L/min in sleep and 1 L/min during the day).

Whole Exome Sequencing (WES) was performed and a total of 100,341 variants were obtained. The first analysis was focused on 55 autoinflammatory genes (Additional file 1: Table S1), but no clinical significant variants were found. However, two clinical relevant heterozygous variants in the ISG15 gene (see Additional file 1: Fig. S1), which codifies for an Ubiquitin-like protein (termed Ubiquitin like modifier ISG15), were identified. The first heterozygous variant c.285del (NM_005101.4, GRCh37(hg19)), also known as c.284del, corresponds to a single nucleotide deletion that is predicted to cause a frameshift in exon 2 (p.Tyr96Thrfs*5) and produces the gain of a stop codon 5 residues downstream from the variant. The second heterozygous variant, c.299_312del (NM_005101.4, GRCh37(hg19)), also known as c.297_313del, corresponds to a 14 nucleotide deletion in exon 2 which is also predicted to produce a frameshift below residue Leu100; p.Leu100Argfs (Fig. 1b). Sanger sequencing and familial segregation were performed confirming that the patient is a compound heterozygous for the above described mutations in ISG15 gene (Fig. 1d). The parents were each heterozygous for one of the reported variants, and the younger brother does not present any of them. All data is consistent with an AR mode of inheritance of *ISG15* deficiency (Fig. 1a). According to ACMG criteria [7], both variants follow the pathogenic criterion: pathogenic very strong (**PVS1**), since they are both null variants, pathogenic moderate 2 (**PM2**) due to their low frequencies (with no reports or very low allele frequency in population databases such as GnomAD); and pathogenic moderate 3 (**PM3**), since we confirmed by Sanger Sequencing that the patient inherited both variants in *trans,* each from one parent. Moreover, it was reported that both variants result in loss of expression and LOF (**PS3**). To accomplish that, Dusan and col. used an in vitro overexpression system in which both variant carrying alleles were compared with the wild type form of ISG15 [8] .

The ISG15 protein is composed of two tandem repeats of ubiquitin-like domains, the N-terminal domain is required for the efficient E3 ligase-mediated transfer of ISG15 from the E2 enzyme UbCH8 to its substrates, although it is dispensable in the activation and transthiolation steps [9]. The two ubiquitin domains of ISG15 play different critical roles in ISGylation, which is unique among ubiquitin and ubiquitin-like modifiers. Both frameshifts variants clearly disrupt the second domain, causing the loss of the C-terminus ending that contains a very conserved amino acid sequence Leu Arg Leu Arg Gly Gly (LRLRGG), corresponding to the ISGylation site and thus triggering the loss of function of the entire protein [10] (Fig. 1b, c). Compared with ubiquitin, which has very high (close to 100% identity) cross-species conservation, ISG15 protein has relatively low cross-species conservation. Its absence in many eukaryotic species suggests that it is not an essential housekeeping gene, leaving more room for its diversification during evolution. Pathogenic variants in *ISG15* gene are a recently defined cause of type I human interferonopathy [11]. Biallelic loss-of‐function variants in *ISG15* were initially described in the context of patients with Immunodeficiency 38 (OMIM: 616126), an AR disorder which confers syndromic Mendelian susceptibility to Mycobacterial diseases (MSMD) and affects about 1 in 50,000 individuals exposed to environmental mycobacteria or BCG vaccine strains [4]. Surprisingly, and despite the antiviral functions of ISG15 described in mice, patients described to date do not present viral susceptibility phenotype. The ISG15-IFN-γ circuit operates principally between granulocytes and NK cells [4, 12]. Elevated ISG expression and cerebral calcification are consistent features in patients with biallelic pathogenic variants in *ISG15*, with a strong neuroradiological overlap with patients with Aicardi–Goutières syndrome (AGS) [13.]

STING-associated vasculopathy with onset in infancy (known as SAVI syndrome) is an early-onset systemic inflammatory phenotype characterized by severe cutaneous vasculopathy and major interstitial lung disease, caused by enhanced sensitivity or ligand-independent (constitutive) activation of a non-nucleic acid receptor component (for example, an adaptor molecule) of the IFN-induced signalling pathway, such as in the case of de novo and inherited pathogenic variants in *TMEM173* gene, which encodes the protein STING (stimulator of interferon genes) [14, 15].

Here, we report a patient that clinically resembles a less severe auto-inflammatory disease, with lung and skin involvement, similar to those observed in other interferonopathies such as SAVI, showing recurrent slow healing skin ulcers and respiratory manifestations, that do not respond adequately to antibiotic treatment nor allow identification of a particular infectious agent. On the other hand, the CT scan of her brain shows basal ganglia calcifications, a common feature present in some autoinflammatory diseases like Chronic Atypical Neutrophilic Dermatosis with Lipodystrophy and Elevated Temperature (CANDLE) and that are most prominent in AGS, but that were also well characterized in patients with Immunodeficiency 38. WES revealed the presence of two protein-truncating pathogenic variants in trans in the *ISG15* gene, both of which eliminate the second ubiquitin-like domain and cause LOF variants.

Our work underscores the strength of WES approach to diagnose immune related disorders with molecular precision, even for isolated (non-familial) cases, leading to the extension of the reported phenotype which now includes recurrent lung disease and cutaneous inflammatory manifestations, without mycobacterial infections. Our findings also highlight the role of *ISG15* as an immunomodulating factor whose LOF mutations result in clinical heterogenous presentations. However it should be noted that the significance of the observed genotype to phenotype association could be limited since we are analyzing a single case. Finally, concerning the molecular mechanism leading to disease, we hypothesize that there might be a factor such as infection, stress or vaccination that may trigger the inflammatory manifestations of the observed multisystemic disease, that initially appeared with skin ulcerations and continued with pulmonary affectation.

## Supplementary information

**Additional file 1: Table S1.** Primary genetic panel studied. Genes related to monogenic autoinflammatory diseases.

**Additional file 2: Table S2**. Patients reported to date with ISG15 deficiency, including ours. IBGC refers to idiopathic basal ganglia calcification.

**Additional file 3: Table S3.** Immune evaluation results. *25th and 75th percentiles of age-related normal values for lymphocyte subpopulations. Cellular immunology laboratory of “Pediatric Hospital Prof. Dr. Juan P. Garrahan”. + Reference values for serum IgA, IgG and IgM according to age, expressed as median ± SD. Ref: Stiehm ER, Fudenberg HH. Serum levels of Immune Globulins in health and disease. Pediatrics 37: 715, 1966.

**Additional file 4: Figure S1.** Sequence alignment view of the BAM file. The two deletions are shown as lines.

## Data Availability

All data generated or analysed during this study are included in this published article [and its supplementary information files].
