# Supplementary material for: Inflammatory cutaneous lesions and pulmonary manifestations in a new patient with autosomal recessive ISG15 deficiency case report
Source: Allergy Asthma Clin Immunol. 2020 Sep 3;16:77. doi: 10.1186/s13223-020-00473-7 (PMC7491304; doi:10.1186/s13223-020-00473-7)
Supplement: Supplementary file 1 — Additional file 1: Table S1. Primary genetic panel studied. Genes related to monogenic autoinflammatory diseases. [file 13223_2020_473_MOESM1_ESM.pdf]

DISEASE	GENE
Spondyloenchondrodysplasia with immune dysregulation	***ACP5***
Inflammatory skin and bowel disease, neonatal, 1	***ADAM17***
Dyschromatosis symmetrica hereditaria AND Aicardi-Goutieres syndrome 6	***ADAR***
Vibratory urticaria	***ADGRE2***
New Autoinflammatory Phenotype	***AGBL3***
Singleton-Merten syndrome 2	***DDX58***
Type I interferon-mediated autoinflammation due to DNase II deficiency	***DNASE2***
Singleton-Merten syndrome 1 AND Aicardi-Goutieres syndrome 7	***IFIH1***
Familial Mediterranean fever	***MEFV***
Hyper-IgD Syndrome/ Mevalonic aciduria/Porokeratosis 3, multiple types	**MVK**
Tumor Necrosis Factor receptor-associated periodic syndrome	***TNFRSF1A, TNFRSF11A***
CINCA syndrome, Familial cold inflammatory syndrome 1, Keratoendothelitis fugax hereditaria, Muckle-Wells syndrome AND Deafness, autosomal dominant 34, with or without inflammation	***NLRP3 (CIAS1, NALP3)***
Familial cold autoinflammatory syndrome 2	***NLRP12 (NALP12)***
Hydatidiform mole, recurrent, 1	***NLRP7***
Blau Syndrome	***NOD2 (CARD15)***
Pyogenic sterile arthritis, pyoderma gangrenosum, and acne	***PSTPIP1***
Majeed Syndrome	***LPIN2***

Interleukin 1 receptor antagonist deficiency	*IL1RN*
Psoriasis 14, pustular	*IL36RN*
Proteasome-associated autoinflammatory syndromes	*PSMG2, PSMA3, PSMB4, PSMB8, PSMB9*
Cherubism	*SH3BP2*
H Syndrome	*SLC29A3*
STING-associated vasculopathy, infantile-onset (SAVI)	*TMEM173*
Autoinflammatory syndrome, familial, Behcet-like	*TNFAIP3*
Polyglucosan body myopathy 1 with or without immunodeficiency	*RBCK1*
Autoinflammation, panniculitis, and dermatosis syndrome	*OTULIN*
Autoinflammation with infantile enterocolitis AND Familial cold autoinflammatory syndrome 4	*NLRC4*
Autoinflammation, antibody deficiency, and immune dysregulation syndrome AND Familial cold autoinflammatory syndrome 3	*PLCG2*
Susceptibility to Psoriasis 15, pustular	*AP1S3*
Pityriasis rubra pilaris AND Psoriasis 2	*CARD14*
ADA2 Deficiency	*CECR1*
IL10/R/RB Deficiency	*IL10, IL10RA, IL10RB*
Autoinflammation with arthritis and dyskeratosis; Palmoplantar carcinoma, multiple self-healing AND Susceptibility to Vitiligo-associated multiple autoimmune disease	*NLRP1*
Proteasome-associated autoinflammatory syndrome 2 AND Keratosis linearis with ichthyosis congenita and sclerosing keratoderma	*POMP*
Autoimmune interstitial lung, joint, and kidney disease	*COPA*
Chilblain lupus, Aicardi-Goutieres syndrome 1, dominant and recessive AND Vasculopathy, retinal, with cerebral leukodystrophy	*TREX1*
Chilblain lupus 2 AND Aicardi-Goutieres syndrome 5	*SAMHD1*
Aicardi-Goutieres syndrome 4	*RNASEH2A*
Aicardi-Goutieres syndrome 2	*RNASEH2B*

Aicardi-Goutieres syndrome 3	***RNASEH2C***
Retinitis pigmentosa and erythrocytic microcytosis	**TRNT1**
Van Esch-O'Driscoll syndrome AND Pigmentary disorder, reticulate, with systemic manifestations, X-linked	***POLA1***
Pseudo-TORCH syndrome 2	***USP18***
Autoinflammatory periodic fever, immunodeficiency, and thrombocytopenia (PFIT)	**WDR1**
Autoinflammation associated with LUBAC deficiency	***SHARPIN/RNF31***
Amyloidosis, primary localized cutaneous, 1	**OSMR**
Multiplex Family with γ-Secretase Spectrum of Autoinflammatory Skin Phenotypes	**NCSTN**
