# Supplementary material for: Inflammatory cutaneous lesions and pulmonary manifestations in a new patient with autosomal recessive ISG15 deficiency case report
Source: Allergy Asthma Clin Immunol. 2020 Sep 3;16:77. doi: 10.1186/s13223-020-00473-7 (PMC7491304; doi:10.1186/s13223-020-00473-7)
Supplement: Supplementary file 2 — Additional file 2: Table S2. Patients reported to date with ISG15 deficiency, including ours. IBGC refers to idiopathic basal ganglia calcification. [file 13223_2020_473_MOESM2_ESM.pdf]

<table>
<thead>
<tr><th>Country of origin</th><th>Patients</th><th>Mendelian susceptibility to mycobacterial diseases (MSMD)</th><th>Viral Infection</th><th>Idiopathic basal ganglia calcification (IBGC)</th><th>Other features</th><th>Reported variants</th></tr>
</thead>
<tbody>
<tr><td rowspan="3">China</td><td>PI female</td><td rowspan="3">No vaccination</td><td rowspan="7">No severe viral infections</td><td>X</td><td>Seizures</td><td rowspan="3">Homozygous nonsense exon 2 variant p.Gln55X</td></tr>
<tr><td>P II female</td><td>X</td><td rowspan="5"></td></tr>
<tr><td>P III female</td><td>X</td></tr>
<tr><td>Turkey</td><td>Female Patient</td><td rowspan="3">BCGitis</td><td>X</td><td>Homozygous nonsense exon 2 variant p.Glu127X</td></tr>
<tr><td rowspan="2">Iran</td><td>PI male</td><td>X</td><td rowspan="2">Homozygous frameshift exon 2 variant p.Leu114fs</td></tr>
<tr><td>PII male</td><td>Along the cerebral falx</td></tr>
<tr><td>Argentina</td><td>Female Patient</td><td>No BCGitis after BCG vaccination at birth</td><td>X</td><td>Respiratory manifestations like recurrent wheezing and lobar pneumonias (seven isolated and unrelated episodes)</td><td>Compound heterozygous variants in exon 2: p.Tyr96fs and p.Leu100fs</td></tr>
</tbody>
</table>
