# Supplementary material for: Inflammatory cutaneous lesions and pulmonary manifestations in a new patient with autosomal recessive ISG15 deficiency case report
Source: Allergy Asthma Clin Immunol. 2020 Sep 3;16:77. doi: 10.1186/s13223-020-00473-7 (PMC7491304; doi:10.1186/s13223-020-00473-7)
Supplement: Supplementary file 3 — Additional file 3: Table S3. Immune evaluation results. *25th and 75th percentiles of age-related normal values for lymphocyte subpopulations. Cellular immunology laboratory of “Pediatric Hospital Prof. Dr. Juan P. Garrahan”. + Reference values for serum IgA, IgG and IgM according to age, expressed as median ± SD. Ref: Stiehm ER, Fudenberg HH. Serum levels of Immune Globulins in health and disease. Pediatrics 37: 715, 1966. [file 13223_2020_473_MOESM3_ESM.pdf]

	1 year and 2 months	3 years and 6 months	4 years and 8 months
**Total Lymphocytes**	7114/mm^3	3746/mm^3	2457/mm^3
**CD3 ***	58%<br>4126/mm^3<br>(3430-4147)	52%<br>1948/mm^3<br>(2054-3169)	58%<br>1425/mm^3<br>(2054-3169)
**CD4 ***	29%<br>2063/mm^3<br>(1718-2550)	26%<br>973/mm^3<br>(1129-1581)	28.7%<br>705/mm^3<br>(1129-1581)
**CD8 ***	27%<br>1920/mm^3<br>(882-1534)	25%<br>936/mm^3<br>(711-1121)	27.5%<br>675/mm^3<br>(711-1121)
**CD3/DR ***	10%	5%	8%
**CD16/56 ***	10.7%<br>761/mm^3<br>(245-503)	5%<br>187/mm^3<br>(246-451)	6.2%<br>152/mm^3<br>(246-451)
**CD19 ***	30%<br>2134/mm^3<br>(756-1260)	41%<br>1535/mm^3<br>(411-658)	34%<br>835/mm^3<br>(411-658)
**IgG $^{+}$**	747 mg/dl<br>(762 +/- 209)	1240 mg/dl<br>(929 +/- 228)	1050 mg/dl<br>(929 +/- 228)
**IgA $^{+}$**	44 mg/dl<br>(50 +/- 24)	50 mg/dl<br>(93 +/- 27)	75 mg/dl<br>(93 +/- 27)
**IgM $^{+}$**	65 mg/dl<br>(58 +/- 23)	49 mg/dl<br>(56 +/-18)	65 mg/dl<br>(56 +/-18)
**Antibody response to vaccines**	Anti-measles IgG positive<br>Anti-rubella IgG positive<br>Anti-HBsAg IgG positive<br>Anti-HAV IgG negative<br>Anti-VZV IgG negative<br>Anti-tetanus toxoid IgG: 0.5 UI/ml<br>Anti-pneumococcal IgG: 25 mg/l		
