# Supplementary figures and images for: Inflammatory cutaneous lesions and pulmonary manifestations in a new patient with autosomal recessive ISG15 deficiency case report
Source: Allergy Asthma Clin Immunol. 2020 Sep 3;16:77. doi: 10.1186/s13223-020-00473-7 (PMC7491304; doi:10.1186/s13223-020-00473-7)

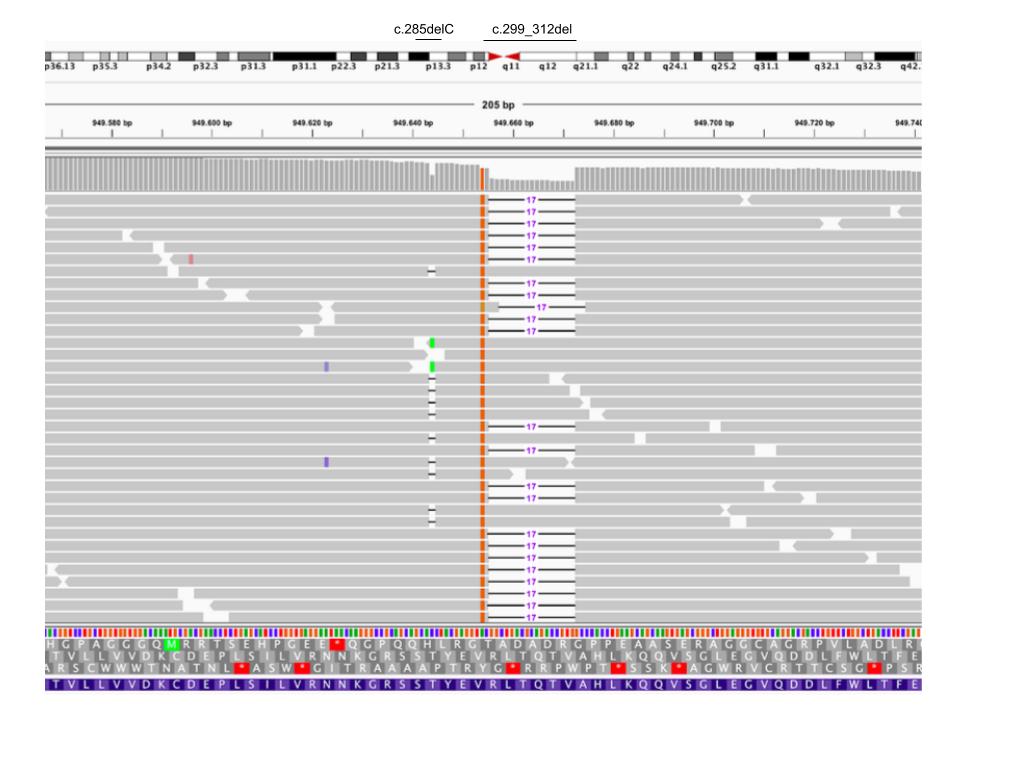

Supplement: Supplementary file 4 — Additional file 4: Figure S1. Sequence alignment view of the BAM file. The two deletions are shown as lines. [file 13223_2020_473_MOESM4_ESM.jpg]
